# Deep Multimodal Sensor Fusion for Lane-Level Traffic Speed Estimation

**DOI:** 10.3390/s26217024

**Published:** 2026-11-06

**Authors:** Kang In Choi, Eun Hak Lee, Seo Jeong Kim, Chang Ho Yu, Youngsun Hong, Sunghwan Jeong

**Affiliations:** 1 AI Application Research Center, Korea Electronics Technology Institute, Jeonju 54853, Republic of Korea; cki921@keti.re.kr (K.I.C.); scott3554@keti.re.kr (S.J.K.); 2 School of Railway Operation Systems, Kyungil University, Gyeongsan 38428, Republic of Korea; eunhak@kiu.ac.kr; 3 Division of Convergence Technology Engineering, Jeonbuk National University, Jeonju 54896, Republic of Korea; redysun@jbnu.ac.kr

**Keywords:** deep multimodal learning, lane-level traffic speed, traffic state estimation, heterogeneous sensors, missing observation estimation, traffic data fusion, route guidance

## Abstract

**Highlights:**

**What are the main findings?**

- The deep multimodal model achieved more accurate and stable lane-level traffic-speed estimation by integrating five heterogeneous sensing sources.
- Multimodal integration reduced dependence on sensor characteristics and improved performance across sensor types, roadway conditions, and sensing environments.

**What are the implications of the main findings?**

- The framework can support missing observation estimation at existing sensor locations by using surrounding links and other available sensing modalities.
- Lane-level multimodal speed estimates can provide more detailed inputs for traffic monitoring and route-cost evaluation, subject to further field validation.

**Abstract:**

This study develops a deep multimodal learning framework for lane-level traffic-speed estimation using five sensing sources: vehicle detection systems (VDS), closed-circuit television (CCTV), light detection and ranging (LiDAR), remote traffic microwave sensors (RTMS), and automatic vehicle classification (AVC). The framework integrates sensor-specific features, road attributes, modality availability, and spatial relationships. Using traffic data from Siheung, Republic of Korea, the proposed model was evaluated against five single-modal baselines. The multimodal model achieved a weighted mean absolute percentage error (WMAPE) below 10% across evaluated time steps, outperforming single-modal models in stability and cross-modal transferability. Improvements were more pronounced in selected multi-lane environments and areas with limited sensing coverage. In route guidance, lane-level multimodal information reduced mean minimum travel time by 7.31% compared with link-level information. These findings demonstrate the potential of multimodal integration for accurate lane-level estimation, recovery of missing sensor observations, and improved route guidance.

## 1. Introduction

Accurate and fine-grained traffic-state information is essential for intelligent transportation systems (ITS), supporting traffic monitoring, congestion management, and proactive traffic control. In the United States, travelers experienced an average of 63 h of congestion delay in 2024, with an estimated annual cost of USD 269 billion [1]. The rapid development of connected and automated vehicles (CAVs) has further increased the demand for detailed traffic information. Conventional traffic monitoring systems primarily measure traffic volume, speed, and occupancy at the link or road-segment level. However, these aggregated measures cannot fully capture traffic variations across individual lanes caused by turning movements, lane changes, and merging and diverging traffic. Lane-level information is therefore important for vehicle maneuvers, including lane selection, merging, car following, and speed control [2,3]. It also supports high-definition maps and digital road infrastructure for automated driving. Recent vision-based systems have further highlighted the importance of microscopic information on vehicle position, speed, and driving behavior [4]. These developments emphasize the need for accurate and reliable lane-level traffic monitoring.

Traffic-state estimation has evolved from conventional link-level analysis toward more detailed spatiotemporal representations. Traffic conditions are characterized by complex spatial and temporal dependencies [5,6], including the influence of distant road links [7] and nonlinear transitions between free-flow and congested conditions [8,9,10]. To capture these dynamics, spatiotemporal learning methods have been widely applied to traffic-speed, travel-time, and traffic-state estimation [11,12,13,14]. More recently, lane-level estimation has received increasing attention because traffic conditions can vary substantially among lanes within the same road section. Existing studies have employed connected vehicles, fixed detectors, trajectory data, roadside video, and deep-learning methods [15,16,17,18]. Gu et al. [16] developed a fusion deep-learning model for lane-level speed prediction, while Liu and Juang [17] proposed a vision-based approach for estimating lane-level traffic flow. Other studies have introduced graph-based and Transformer-based models to capture interactions among individual lanes [19,20,21]. These advances demonstrate the potential of lane-level modeling to represent localized traffic dynamics that are often overlooked by conventional link-level approaches.

Despite these advances, the accuracy and coverage of lane-level traffic information remain dependent on the sensing technologies. Different sensors capture different traffic characteristics and provide complementary information [22,23,24]. Fixed detectors such as vehicle detection systems (VDS) measure traffic volume, speed, and occupancy at specific locations, while closed-circuit television (CCTV) supports vehicle detection, lane-use analysis, and trajectory extraction [17,22]. LiDAR provides high-resolution three-dimensional information for vehicle detection, tracking, and trajectory reconstruction [25,26]. Remote traffic microwave sensors (RTMSs) use radar technology to monitor traffic conditions across multiple lanes [23,24], whereas automatic vehicle classification (AVC) systems provide vehicle counts and classification information. However, these technologies differ in spatial coverage, resolution, measurement characteristics, and sensitivity to environmental conditions. Their performance may vary with weather, occlusion, traffic density, and installation geometry. Consequently, no single sensing technology can provide complete and reliable lane-level traffic information across different roadway environments.

To address the limitations of individual sensors, multi-source data fusion has emerged as an approach to traffic-state estimation [27]. Early studies incorporated probe-vehicle observations and fixed or mobile sensor data into traffic-state estimation frameworks [28,29,30]. Recent research has extended these approaches to heterogeneous detectors, vehicle trajectories, connected vehicles, and roadside sensing systems [31,32,33,34]. Liu et al. [18] integrated connected-vehicle, probe-vehicle, and fixed-detector data for lane-level freeway traffic-state estimation, while Zhang et al. [32] developed a heterogeneous sensor fusion framework for freeway traffic-state estimation. Deep-learning methods have been applied to capture complementary information across sensing sources [33,34]. Min et al. [35] proposed a deep multimodal model combining dedicated short-range communication (DSRC) and VDS data for traffic-speed estimation. Although these studies demonstrate the benefits of multi-source integration, many approaches remain focused on link-level traffic states or rely on a limited combination of sensing technologies.

A challenge in multi-source traffic estimation is the integration of sensor observations with spatial, temporal, and measurement characteristics. Heterogeneous sensors may introduce inconsistencies in reporting intervals, detection locations, and measurement definitions, making cross-sensor calibration and data alignment essential. Zhao et al. [36] proposed a graph-matching framework for spatiotemporal calibration of roadside sensors, while Maiti and Leclercq [37] developed a machine-learning approach to estimate spatial mean speeds from local sensor measurements. Jiao et al. [34] addressed differences in sensor reporting frequencies through interpolation before applying deep-learning models. Maiti et al. [38] emphasized the importance of bias correction and systematic data fusion for consistent traffic-state representation. Thus, effective multimodal fusion requires not only the integration of multiple data sources but also consistent spatiotemporal alignment and consideration of sensor-specific measurement characteristics.

Building on these findings, this study develops a deep multimodal learning framework for lane-level traffic speed estimation using real-world sensing data collected in Siheung, Republic of Korea. Five sensing sources are integrated: VDS, CCTV, LiDAR, RTMS, and AVC. The heterogeneous data are preprocessed and temporally aligned, and sensor-specific features are extracted and fused through a deep multimodal architecture. The proposed framework makes three main contributions. First, it integrates five sensing modalities to capture complementary traffic information and reduce dependence on individual sensing sources. Second, it estimates traffic speed at the lane level, providing more detailed representations of localized traffic conditions than conventional link-level approaches. Third, it estimates lane-level speed at target sensor locations whose observations are unavailable by leveraging concurrent information from surrounding links and other available sensing sources. Together, these contributions provide a framework for improving the spatial coverage and resolution of traffic-state estimation in heterogeneous sensing environments.

## 2. Data

### 2.1. Overall Data Description

**Figure 1:**
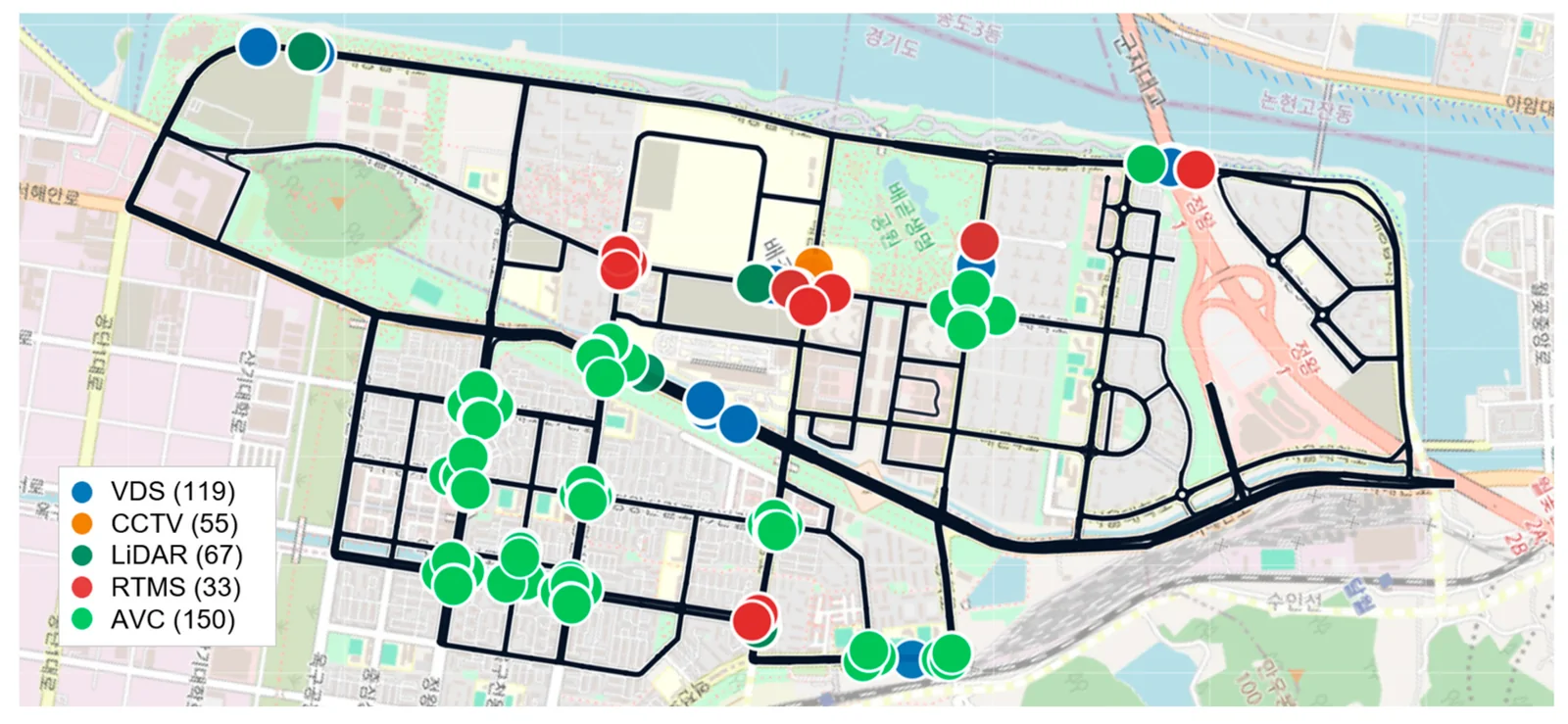
The study area and locations of the five sensing systems. Black lines denote study links; colored markers identify sensor types. Shading and Korean place names belong to the basemap.

This study used traffic sensing data collected from the road network in Siheung, Republic of Korea. The study area represents a typical urban traffic environment with residential, commercial, business, park, and waterfront areas. The network consists of 821 links and 2035 lanes, with a total centerline length of approximately 117.5 km. It includes 413 intersection nodes, including 3 signalized intersections, 400 priority intersections, and 8 terminal nodes. Two-lane links are the most common, and roads with two to five lanes are relatively well distributed throughout the network. The network includes both major arterial roads and local streets. Five sensing systems are distributed throughout the network. This configuration allows the study to compare differences in traffic observations across sensor types and roadway environments. The study area is also well suited for analyzing lane-level traffic conditions, including speed variations and differences among individual lanes. Figure 1 presents the study area and locations of the five sensing systems.

Specifically, the dataset consists of five infrastructure-based sensing sources: vehicle detection system (VDS), closed-circuit television (CCTV), LiDAR, remote traffic microwave sensor (RTMS), and automatic vehicle classification (AVC). These sensing systems are installed at different roadway locations and provide traffic information through different measurement principles. The use of multiple sensing sources allows traffic conditions to be observed from several perspectives instead of relying on a single detector type.

The five datasets differ in their data structure, measurement variables, spatial coverage, and temporal resolution. VDS mainly provides conventional traffic variables such as speed, volume, and occupancy. CCTV and LiDAR provide more detailed information on individual vehicle movements and lane-specific traffic conditions. RTMS measures traffic conditions using microwave-based roadside sensing, and AVC provides traffic counts and vehicle classification information. These differences provide complementary information for lane-level traffic-state estimation.

To construct a common dataset for multimodal learning, the raw data from each sensing source were first processed independently. The observations were then temporally aligned and matched with the corresponding roadway and lane information. A common temporal structure was used to integrate heterogeneous sensing data. The processed dataset was subsequently used to train both the proposed deep multimodal model and the individual single-source models.

### 2.2. Vehicle Detection System (VDS)

A VDS is a fixed traffic sensor that continuously measures traffic conditions at designated roadway sections. It typically provides speed, traffic volume, and occupancy in real time. VDS is particularly suitable for mainline and arterial road sections, where traffic flow is continuous and repeated patterns can be observed over time. Its main strength is the stable collection of continuous traffic-flow information. In the Siheung network, VDS detectors are installed at 119 locations. The detectors are mainly distributed along major roadway sections and arterial approaches. VDS therefore serves as a reference source for capturing recurrent traffic patterns, time-of-day congestion, and link-level speed variations across the network. This makes VDS well suited for representing the baseline traffic condition of major roadway sections.

### 2.3. Closed-Circuit Television (CCTV)

CCTV is a non-intrusive visual sensor that continuously observes traffic conditions through roadway video. It can capture vehicle movements, traffic volume, speed, lane use, and local congestion over a wide field of view. CCTV is useful at intersections, connecting roads, and locations with complex traffic interactions. It can observe turning movements and localized traffic changes that are difficult to capture with point-based detectors. A total of 55 CCTV locations are included in the Siheung network. The cameras are mainly located near urban intersections and major connecting roads. CCTV therefore provides complementary information on localized traffic conditions, including signal-related fluctuations, turning traffic, and irregular congestion. Its visual coverage is useful for observing short-term and localized changes around intersection areas.

### 2.4. LiDAR

LiDAR is a spatial sensing technology that uses laser pulses to measure the distance and position of surrounding objects in real time. It can identify individual vehicles and derive detailed information on their movement, speed, and trajectories from high-resolution three-dimensional observations. LiDAR is particularly suitable for lane-level and microscopic traffic observation, because it can distinguish vehicle movements and spatial relationships across adjacent lanes. The Siheung network includes 67 LiDAR locations. The sensors are installed along major urban links and near selected intersections. Their main role is to capture detailed lane-level movements, including lane changes, vehicle spacing, merging, and localized traffic disturbances. LiDAR therefore provides a more spatially detailed observation source than conventional fixed detectors. This capability allows LiDAR to capture fine spatial differences that are difficult to represent using aggregated detector measurements.

### 2.5. Remote Traffic Microwave Sensor (RTMS)

An RTMS is a roadside radar-based sensor that detects vehicle presence and movement using microwave signals. It continuously measures traffic variables such as speed and volume across multiple lanes. RTMS is particularly suitable for continuous multilane monitoring on major roadway sections. It is also less sensitive to lighting conditions than vision-based sensors. The Siheung network includes 33 RTMS locations. RTMSs are mainly installed along major roads where continuous multilane monitoring is required. Their role is to capture stable traffic-flow information across several lanes under different operating conditions. This makes RTMS useful for maintaining stable traffic observations under varying environmental conditions.

### 2.6. Automatic Vehicle Classification (AVC)

AVC is a traffic detection system that continuously identifies and classifies passing vehicles by type. It also collects traffic-flow information about traffic volume and speed. Its main advantage is that it provides information on traffic composition as well as overall traffic conditions. This makes AVC useful for identifying differences between passenger-car traffic and flows with a higher proportion of buses or heavy vehicles. The Siheung network includes 150 AVC locations, representing the largest spatial coverage among the five sensing sources. AVC sensors are widely distributed across arterial roads and local road sections. They therefore provide broad network coverage and capture spatial variations in traffic demand. Their vehicle-classification capability also provides additional information on traffic composition that cannot be obtained directly from speed-oriented sensors alone.

### 2.7. Data Processing

The raw data collected from VDS, CCTV, LiDAR, RTMS, and AVC sensors were organized into a common format. Sensor identifiers were standardized across the five modalities, and each sensor record was matched to its corresponding road link using the sensor catalog and network geometry. The sensor data were supplied at five-minute intervals with explicit lane identifiers. Records were aligned using timestamps representing the same five-minute interval, and lane assignments were based on the lane numbers provided in the source data. The study period extended from 5 March 2026 to 2 April 2026. Speed was used as the main traffic variable for all sensor types.

The dataset was divided chronologically into training, validation, and test sets comprising the first 60%, subsequent 20%, and final 20% of the time steps, respectively. All observations sharing the same timestamp were assigned to the same subset. The training set was used for model fitting, the validation set for model selection, and the test set exclusively for final performance evaluation. Identical temporal splits were applied to the deep multimodal model and all single-modal baselines. With 7986 time steps in total, the training, validation, and test sets contain approximately 4792, 1597, and 1597 five-minute time steps, corresponding to approximately 16.6, 5.5, and 5.5 days, respectively.

After temporal alignment and chronological splitting, missing input values were imputed using ARIMA models fitted exclusively to the training data. For validation and testing, imputation proceeded chronologically using only preceding observations, with model parameters held fixed. The original observation masks were retained, and evaluation metrics were calculated only for originally observed target values. ARIMA models were fitted separately for each sensor, and imputed values were used only as model inputs and never as evaluation targets. All normalization parameters were likewise estimated from the training set only.

For model input construction, the processed observations were transformed into link-level features by combining temporal traffic measurements with the spatial information of each sensor. This process ensured a common temporal index across all modalities while retaining their sensor-specific characteristics. The final dataset contains 7986 time steps and 424 sensor sites. The five modalities differ in installation density, measurement principle, and spatial coverage. Table 1 presents the descriptive statistics of the processed data for each sensor type. AVC accounted for the largest number of sites (150), followed by VDS (119), whereas RTMS had the fewest sites (33). Mean speeds ranged from 8.08 km/h for RTMS to 60.51 km/h for AVC, highlighting the variation in recorded traffic conditions across sensing sources.

## 3. Methods

### 3.1. Research Framework

The overall framework consists of three main stages: data preprocessing, model training, and performance evaluation. Heterogeneous observations from VDS, CCTV, LiDAR, RTMS, and AVC are first standardized, temporally aligned, and organized into a common sensor–time structure. The processed data are then used to construct sensor-specific inputs for model training. The model was implemented in Python 3.12.14 using PyTorch 2.7.1; inference was benchmarked with CUDA 12.8.

The proposed model learns separate representations from each sensing source and integrates them into a shared latent space. This structure captures complementary traffic information across sensors with different measurement principles and spatial coverage. The fused representation is then used to estimate lane-level traffic speed.

**Figure 2:**
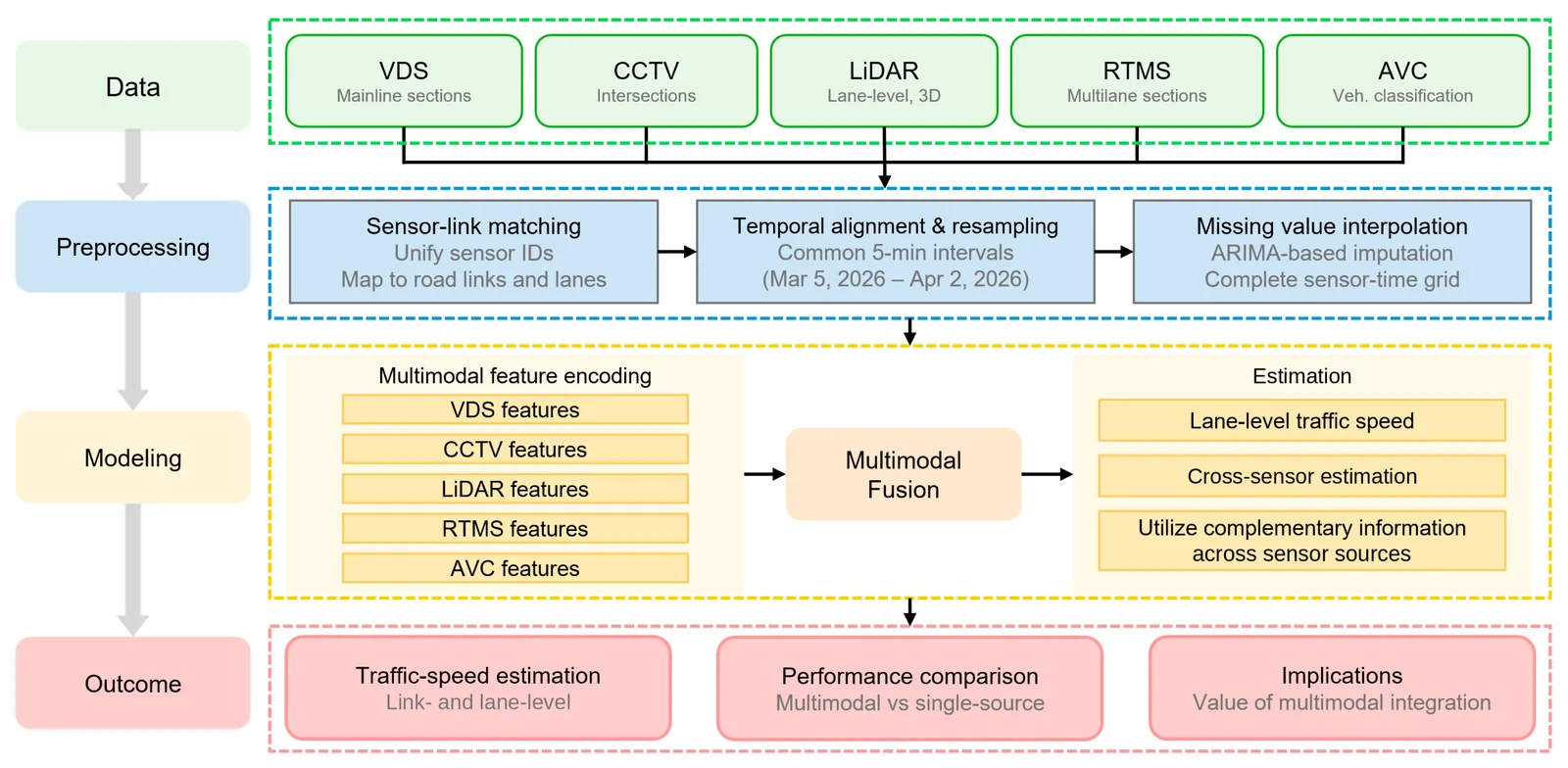
Overall framework of the deep multimodal learning model. Colors and dashed outlines group the workflow stages; solid boxes identify their components, and arrows indicate processing order.

For evaluation, the deep multimodal model is compared with corresponding single-modal baseline models trained independently using VDS, CCTV, LiDAR, RTMS, and AVC data. This comparison quantifies the benefit of multimodal integration and assesses whether combining heterogeneous sensing sources improves the accuracy and robustness of lane-level traffic-speed estimation. The overall process is illustrated in Figure 2.

### 3.2. Problem Definition

The objective of this study is to estimate lane-level traffic speed using heterogeneous infrastructure sensing data. Each estimation sample is defined by a specific target sensor location, its associated road link and lane, and a corresponding time step. Traffic observations from VDS, CCTV, LiDAR, RTMS, and AVC are organized within the same temporal and spatial framework. The model uses these observations to estimate the speed recorded at each target sensor location. In this way, the estimation task focuses on local lane-level traffic conditions rather than an aggregated traffic state for the entire road link.

Formally, let $x_{t,i}^{\left(m\right)}$ denote the observation of modality *m* at sensor link *i* at time *t*, and let 𝒟*_q_* denote the set of donor sensor links available for target sensor *q*, excluding the target sensor cell itself. The estimation target and the information set available to the model are defined as

(1) $${\hat{y}}_{t,q}=F\left(\left\{x_{t,i}^{\left(m\right)}|i\in D_{q},\;m=1,\ldots ,M\right\},\;s_{q},\;\left\{p_{q,i}|i\in D_{q}\right\}\right)$$

where **s***_q_* denotes the static road attributes of the target link and **p***_q_*_,*i*_ the target–donor spatial pair features. Only observations recorded at the same time *t* are used, and no observation from the target sensor or from any other time step is included. The task is therefore a contemporaneous spatial estimation of the speed at the target sensor, that is, the reconstruction of an unavailable observation from other concurrently operating sensors, rather than a forecast of future traffic conditions.

Ground truth is defined as the speed observed by each individual target sensor (VDS, CCTV, LiDAR, RTMS, or AVC), rather than the average across sensors on the same road link. This preserves sensor-specific and lane-specific variations. Accordingly, estimates represent the speed reported by the target sensor, including its measurement characteristics, rather than a sensor-independent traffic speed.

Under this formulation, the deep multimodal model estimates the speed of a target sensor location using information collected from all available sensing modalities. The model can therefore learn traffic patterns from sensors with different spatial coverage and measurement characteristics. Information from one sensing source can complement conditions that are less clearly represented by another source. This enables the model to exploit complementary information across sensor types while preserving the original observation characteristics of each target sensor.

Three related tasks can be distinguished: (i) reconstruction of an occasional missing measurement at an existing sensor, (ii) estimation during a temporary sensor failure, and (iii) estimation at a road or lane where no sensor has ever been installed. Because each target sensor also appears as a supervised target during the training period and its static attributes remain available, the experiments in this study correspond to task (i) and, only partially, to task (ii). Estimation at never-instrumented locations (task (iii)) requires holding out complete sensors, links, or regions from training and is beyond the scope of the present evaluation.

### 3.3. Deep Multimodal Learning Model

**Figure 3:**
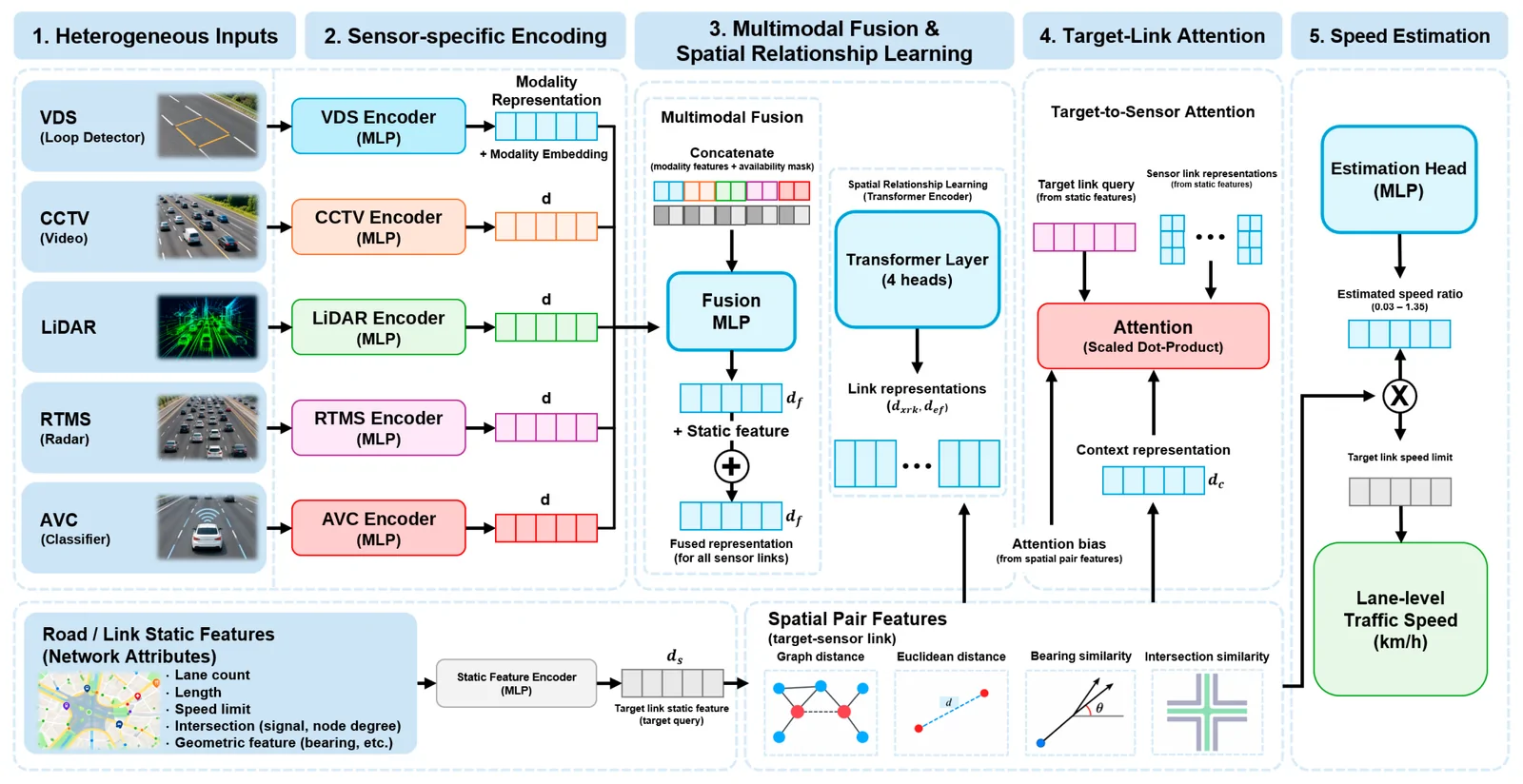
Architecture of the deep multimodal learning model for lane-level speed estimation. Colors distinguish sensor inputs. Arrows show data flow, dashed outlines group related operations, and the plus and multiplication symbols indicate addition and speed scaling, respectively.

The model is designed to preserve the characteristics of each sensing modality while learning their complementary relationships. In the first stage, VDS, CCTV, LiDAR, RTMS, and AVC observations are processed through separate modality-specific encoders. This allows each sensor type to be represented independently before fusion. The encoded features are then combined with sensor-availability information and static road attributes to construct a unified representation for each sensor link. A Transformer-based spatial learning module is subsequently used to capture relationships among sensor links across the network. For each target location, the model applies target-to-sensor attention to identify the most relevant donor links based on both learned representations and spatial relationships. The resulting context is finally passed through an estimation head to estimate the target traffic speed. Figure 3 illustrates the overall architecture of the proposed model.

For each sensor modality, the dynamic traffic features are first transformed through an independent modality-specific encoder. The input for each sensor-link cell consists of eight features: normalized speed ratio, speed ratio variability, normalized traffic volume, normalized occupancy, sensor position, intersection-approach information, direct-mapping status, and sensor-count information. These features represent both traffic conditions and sensor-specific characteristics. Each encoder consists of fully connected layers with Layer Normalization and GELU activation. A learnable modality embedding is also added so that the model can distinguish VDS, CCTV, LiDAR, RTMS, and AVC representations. The encoded representation for modality *m* at time *t* and sensor link *i* is expressed as

(2) $$h_{t,i}^{\left(m\right)}=f_{m}\left(x_{t,i}^{\left(m\right)}\right)+e^{\left(m\right)},$$

where $x_{t,i}^{\left(m\right)}$ denotes the dynamic input features, *f_m_*(·) is the modality-specific encoder, and **e**^(*m*)^ is the learnable modality embedding. Separate encoders are used for the five sensing sources before multimodal fusion.

The encoded modality features are combined with sensor-availability information and passed through the multimodal fusion network. Static road attributes are processed separately through a static feature encoder and added to the fused representation. The static variables include lane count, link length, speed limit, road priority, link coordinates, bearing information, node degree, intersection-related variables, signalized approach and departure indicators, and a midblock indicator. The fused representation is defined as

(3) $${\tilde{f}}_{t,i}=g_{\mathrm{fusion}}\left(\left[a_{i}^{\left(1\right)}h_{t,i}^{\left(1\right)};\ldots ;a_{i}^{\left(M\right)}h_{t,i}^{\left(M\right)};a_{i}\right]\right)+g_{\mathrm{static}}\left(s_{i}\right),$$

where $a_{i}^{\left(m\right)}$ indicates whether modality *m* is available at link *i*, **a***_i_* is the modality-availability vector, and **s***_i_* denotes the static road features. This structure allows the model to jointly represent traffic conditions, sensor characteristics, and roadway attributes.

The fused sensor-link representations are then passed through a two-layer Transformer encoder with four attention heads. This stage learns spatial relationships among sensor-equipped links and allows each link representation to incorporate information from other parts of the network. The spatial interaction is not restricted to directly adjacent links. Instead, the model evaluates the relationship between each target link and available donor links using both learned representations and explicit network features. Four pairwise spatial features are used: graph proximity, Euclidean proximity, bearing similarity, and intersection similarity. Graph proximity is derived from the number of network hops between the target and donor links, and Euclidean proximity reflects their physical distance. Bearing similarity represents directional consistency, and intersection similarity indicates whether the two links have similar intersection characteristics.

For a target link *q*, the static road representation is used to generate a target query. The encoded donor-link representations provide the corresponding keys. The pairwise spatial features are incorporated as an additional attention bias. The attention score and normalized weight are defined as follows:

(4) $$e_{t,q,i}=\frac{q_{q}^{⊤}k_{t,i}}{\sqrt{d_{h}}}+g_{\mathrm{pair}}\left(p_{q,i}\right),\;\alpha_{t,q,i}=\frac{\exp(e_{t,q,i})}{\sum_{j\in D_{q}}\exp(e_{t,q,j})},$$

where **q***_q_* is the target-link query, **k***_t_*_,*i*_ is the encoded representation of donor link *i*, **p***_q_*_,*i*_ contains the four spatial pair features, and 𝒟*_q_* denotes the set of available donor sensor links. This mechanism allows the model to assign greater weight to donor links that are more relevant to the target based on both traffic patterns and network structure. In addition, *d_h_* is the dimension of each attention head, and *g_pair_*(·) is a multilayer perceptron that converts the pair features into an attention bias.

The attention weights are used to aggregate information from the donor links. The model generates an attention-weighted context representation and an attention-weighted donor speed ratio. A global donor ratio is also used as a broader traffic-state reference.

(5) $$c_{t,q}=\sum_{i\in D_{q}}\alpha_{t,q,i}H_{t,i},r_{t,q}^{\mathrm{mix}}=\sum_{i\in D_{q}}\alpha_{t,q,i}r_{t,i},$$

(6) $${\hat{r}}_{t,q}=g_{\mathrm{head}}\left(\left[r_{q};c_{t,q};r_{t,q}^{\mathrm{mix}};r_{t}^{\mathrm{global}}\right]\right),{\hat{v}}_{t,q}={\hat{r}}_{t,q}v_{q}^{\mathrm{limit}}.$$

where **c***_t_*_,*q*_ represents the context obtained from the donor links, $r_{t,q}^{\mathrm{mix}}$ is the attention-weighted donor speed ratio, and $r_{t}^{\mathrm{global}}$ is the global donor reference. The estimation ratio ${\hat{r}}_{t,q}$ is converted into traffic speed using the speed limit of the target link. This formulation allows traffic information from multiple sensing modalities and spatially related links to contribute to the estimation of a specific target sensor location. The model is trained using a combined objective function consisting of Smooth L1 loss and a relative-error term. In addition, **H***_t_*_,*i*_ denotes the output representation of donor link *i* from the Transformer encoder, *r_t_*_,*i*_ is the observed speed ratio of donor link *i*, **r***_q_* is the static representation of target link *q*, *g_head_*(·) is the multilayer perceptron of the estimation head, and $v_{q}^{\mathrm{limit}}$ is the speed limit of target link *q*.

(7) $$L=L_{\mathrm{SmoothL}1}+\lambda \frac{1}{N}\sum_{n=1}^{N}\frac{|{\hat{r}}_{n}-r_{n}|}{\max(r_{n},\epsilon )}.$$

In the implemented model, *λ* = 0.08, and the Smooth L1 parameter is set to *β* = 0.06. The constant *ϵ* in Equation (7) prevents division by zero. During training and evaluation, the target sensor cell is excluded from the multimodal input to prevent direct use of the observation being estimated. The deep multimodal model uses all remaining sensing modalities. The single-modal models follow the same estimation framework but retain only their corresponding sensing source. This setup enables a direct comparison between multimodal and single-modal learning and quantifies the performance gain obtained from heterogeneous sensor integration.

### 3.4. Single-Modal Baseline Models

To evaluate the contribution of multimodal integration, five single-modal baseline models were constructed for VDS, CCTV, LiDAR, RTMS, and AVC. Each baseline follows the same target definition and estimation framework as the deep multimodal model. The difference is that only one modality is retained as the input source. For a given modality m, the donor set is restricted to sensor links belonging to that modality:

(8) $$D_{q}^{\left(m\right)}=\left\{i|a_{i}^{\left(m\right)}=1,\;i\neq q\right\},$$

where $D_{q}^{\left(m\right)}$ denotes the donor set for modality m, $a_{i}^{\left(m\right)}$ indicates the availability of modality mat sensor link i, and q is the target sensor location. The target observation is excluded from the donor set in the same manner as in the deep multimodal model.

The single-modal estimation can then be represented as follows:

(9) $${\hat{v}}_{t,q}^{\left(m\right)}=F^{\left(m\right)}\left({\left\{x_{t,i}^{\left(m\right)}\right\}}_{i\in D_{q}^{\left(m\right)}},s_{q},p_{q,i}\right),$$

where F^(m)^(·) denotes the single-modal model, $x_{t,i}^{\left(m\right)}$ represents the traffic observations from modality m, ***s***_q_ denotes the target-link static features, and ***p***_q,i_ represents the spatial relationship between the target and donor links.

Accordingly, five baseline models are evaluated: single-VDS, single-CCTV, single-LiDAR, single-RTMS, and single-AVC. The same target definition, training procedure, and evaluation conditions are maintained across all models. This setup allows the performance difference between the deep multimodal model and single-modal baselines to be attributed to additional information obtained from heterogeneous sensor integration.

### 3.5. Performance Evaluation Metrics

The performance of the proposed deep multimodal model and the five single-modal baseline models was evaluated using three error metrics: weighted mean absolute percentage error (WMAPE), mean absolute error (MAE), and root mean squared error (RMSE). These metrics evaluate relative estimation error, average absolute deviation, and sensitivity to large errors. WMAPE measures overall relative estimation error, MAE represents the average absolute estimation error, and RMSE assigns greater weight to large estimation errors. The three metrics are calculated as follows:

(10) $$\mathrm{WMAPE}=\frac{\sum_{n=1}^{N}|y_{n}-{\hat{y}}_{n}|}{\sum_{n=1}^{N}|y_{n}|}\times 100,$$

(11) $$\mathrm{MAE}=\frac{1}{N}\sum_{n=1}^{N}|y_{n}-{\hat{y}}_{n}|$$

(12) $$\mathrm{RMSE}=\sqrt{\frac{1}{N}\sum_{n=1}^{N}{\left(y_{n}-{\hat{y}}_{n}\right)}^{2}}$$

where y_n_ and ${\hat{y}}_{n}$ denote the observed and estimated traffic speeds, respectively, and Nis the number of observations. WMAPE provides a scale-independent measure of overall estimation error and was used as the primary evaluation metric in this study.

Lower values of WMAPE, MAE, and RMSE indicate better estimation performance. The same metrics were applied to the deep multimodal model and all single-modal baselines to ensure a consistent comparison under identical evaluation conditions.

## 4. Results

### 4.1. Overall Performance

The overall performance of the deep multimodal model and five single-modal models across evaluation time steps is presented in Table 2. The deep multimodal model achieved lower estimation errors across most time steps and metrics. Its WMAPE ranged from 8.22% to 9.71%, remaining below 10%, while MAE ranged from 5.86 to 6.70. These results indicate relatively stable estimation performance across the evaluated time steps. Here, the time step denotes the temporal aggregation interval at which the input and target speeds were aggregated and evaluated (5 min, 15 min, 30 min, 1 h, and 2 h), starting from the original 5 min data; it does not represent a forecast horizon.

Performance varied among the single-modal models. Single-VDS performed best at shorter time steps, while single-AVC and single-RTMS achieved low errors. Single-CCTV showed the highest errors, with WMAPE exceeding 20% at all time steps. Single-LiDAR produced higher errors than VDS, RTMS, and AVC. These differences suggest that observation characteristics and spatial coverage influence estimation accuracy, with no single modality performing best across all metrics and time steps.

The deep multimodal model outperformed the best single-modal model in WMAPE at every time step. The improvement was small at the shortest time step but became more pronounced at intermediate and longer time steps. MAE showed a similar pattern, suggesting that complementary sensing information helps maintain accuracy across temporal evaluation settings. RMSE showed one exception: at the 5 min time step, single-VDS achieved 7.32, compared with 7.70 for the deep multimodal model. At subsequent time steps, the deep multimodal model achieved lower RMSE. This pattern highlights the value of multimodal integration in maintaining consistent performance across temporal settings, even when an individual sensor performs better on a particular metric.

Overall, multimodal integration combined complementary information and reduced dependence on a single technology. Its benefit was consistent accuracy across the time steps and metrics, supporting its use in networks with heterogeneous sensor coverage.

An ablation analysis examined the contributions of the Transformer, spatial attention bias, and multimodal fusion design. As shown in Table 3, the full model achieved the lowest WMAPE at 8.92%. Removing the Transformer increased WMAPE to 9.49%, corresponding to a deterioration of 0.57 percentage points. Removing the spatial bias produced a smaller increase to 9.07%, or 0.15 percentage points. The simple-fusion variant showed the highest WMAPE at 11.44%, exceeding the full model by 2.52 percentage points. Each ablated variant therefore produced higher estimation error than the full model, although the magnitude differed across components.

The largest performance loss occurred with simple fusion, highlighting the value of the proposed fusion design for integrating heterogeneous sensing information. This result suggests that estimation accuracy depends on how sensor-specific representations are combined, beyond the availability of multiple sensing sources. The Transformer also contributed to lower estimation error, supporting the benefit of learning spatial relationships among sensor-equipped links. Spatial attention bias provided a smaller numerical improvement, suggesting that explicit network features can complement the learned relationships. Together, these results support the combined architecture, with the fusion design contributing the largest observed improvement within the evaluated setting.

To evaluate computational feasibility for 5 min traffic updates, the full model was benchmarked across 821 network links, generating 4105 modality-conditioned estimates per update. Measurements were collected over 100 input snapshots after 10 warm-up runs, using a batch size of 256 and FP16 inference on an NVIDIA RTX 5070 Ti GPU. As shown in Table 4, mean GPU inference latency was 35.68 ms, with a 95th-percentile latency of 37.77 ms. Processing latency, including query construction, CPU–GPU transfers, inference, and output speed conversion, averaged 47.98 ms, with a 95th percentile of 51.08 ms. These measurements indicate that the inference workload is comfortably within the 5 min update interval. However, timing began from precomputed input features and excluded raw-data preprocessing, model loading, and file storage; it therefore does not establish end-to-end deployment latency or lane-level inference performance.

### 4.2. Performance by Sensor Modality

As shown in Table 5, the deep multimodal model achieved lower WMAPE than the matched single-modal baselines for CCTV and AVC. WMAPE decreased from 21.67% to 20.76% for CCTV and from 11.38% to 10.20% for AVC, corresponding to numerical gains of 0.91 and 1.18 percentage points, respectively. These improvements suggest that information from additional sensing modalities can provide complementary value for these target sensors.

For VDS, LiDAR, and RTMS, the deep multimodal model achieved WMAPE values of 2.76%, 9.60%, and 8.21%, respectively. The absolute differences from the matched single-modal baselines remained within 0.20 percentage points. Thus, a single multimodal model produced estimates close to those of the corresponding sensor-specific models across these three targets while achieving numerical improvements for CCTV and AVC. Across the five target sensor types, the macro-averaged WMAPE was 10.31% for the deep multimodal model and 10.63% for the matched single-modal models, whereas the sample-weighted (pooled) results are reported in Table 2.

As a supplementary comparison, the deep multimodal model achieved lower WMAPE than the average single-modal baseline across all five target sensor types, with the largest reduction observed for VDS at 10.06 percentage points. Together, these findings highlight the potential of a unified multimodal framework to support traffic-speed estimation across heterogeneous sensing environments and provide complementary benefits for selected target modalities.

### 4.3. Cross-Modal Analysis

**Figure 4:**
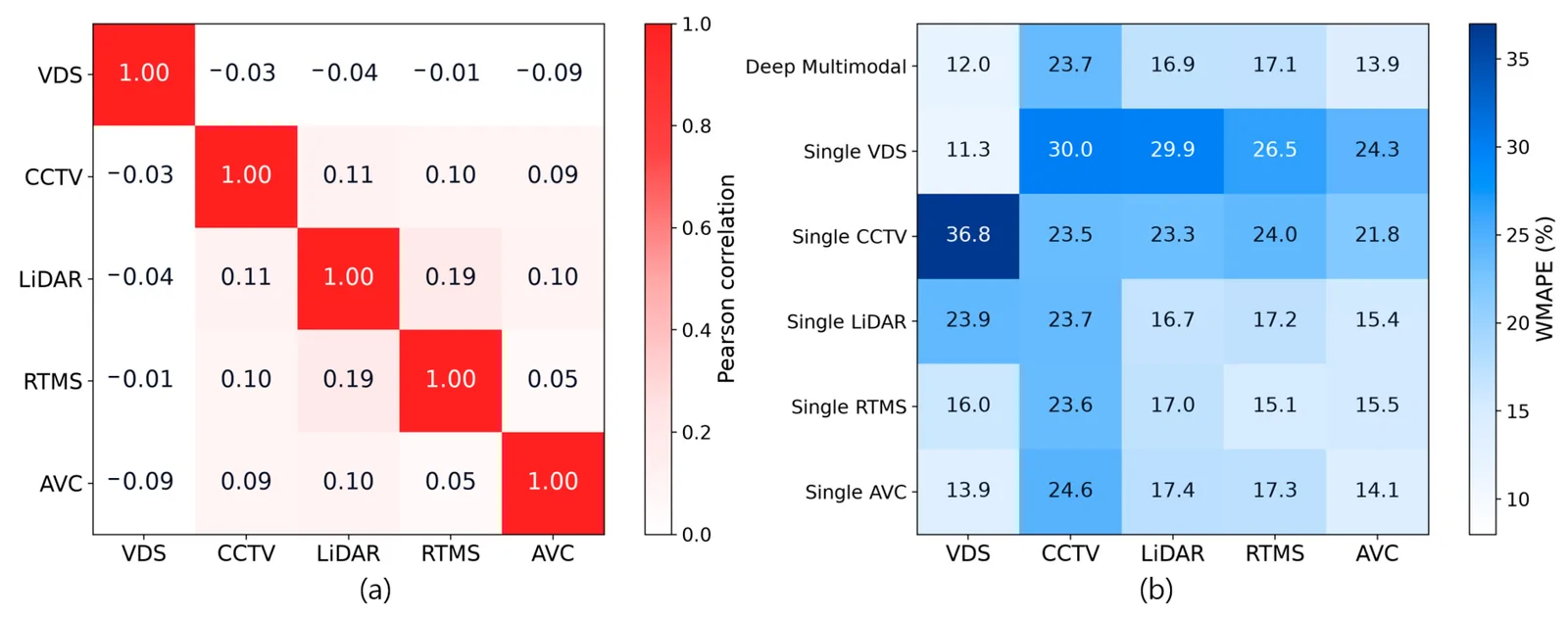
Cross-modal relationships and estimation performance: (**a**) Pearson correlation of observed speeds across sensing modalities and (**b**) WMAPE across target sensor modalities.

To examine cross-modal transferability, the relationships among sensing modalities and the estimation performance across different target sensors were analyzed. Figure 4a presents the Pearson correlations between observed speeds from the five sensing modalities. The correlations are generally low, ranging from −0.09 to 0.19. The highest correlation is observed between LiDAR and RTMS at 0.19. VDS shows weak negative correlations with the other sensing sources. These low correlations indicate that the observed speeds differ substantially across modalities. However, they may reflect differences in deployment location, road class, speed limit, traffic regime, aggregation rule, and systematic sensor bias, as well as complementary traffic information. Because the correlation analysis was not controlled for these factors, it is interpreted descriptively.

Figure 4b compares the cross-modal estimation performance of the deep multimodal and single-modal models. Each single-modal model generally performs best when the target sensor belongs to the same modality. Performance decreases when the model is applied to other sensing sources. For example, single-VDS achieves a WMAPE of 11.3% for the VDS target. The error increases to 30.0% for CCTV and 29.9% for LiDAR. Single-CCTV shows a similar pattern, with 23.5% for the CCTV target and 36.8% for VDS. These results indicate limited transferability across sensor modalities.

The degree of cross-modal transfer also differs by sensing source. Single-LiDAR and single-RTMS show relatively better transfer to several non-matching targets than single-VDS or single-CCTV. Single-LiDAR achieves WMAPE values of 17.2% and 15.4% for RTMS and AVC targets, respectively. Single-RTMS also maintains relatively low errors for LiDAR and AVC targets. This suggests that some sensing modalities share more transferable traffic information despite weak direct speed correlations.

In contrast, the deep multimodal model maintains more balanced performance across all target sensor types. A matched single-modal model can slightly outperform the multimodal model for its own target modality, as also shown in Table 5; however, this advantage is limited to its own sensing source. The deep multimodal model avoids the large error increase observed when single-modal models are applied to non-matching sensor types.

Overall, the results show that multimodal integration provides robust estimation across heterogeneous target sensor types. The modality-dependent errors of the single-modal models further support the value of heterogeneous sensing for network-wide traffic-speed estimation.

### 4.4. Lane-Level Performance Analysis

Lane-level performance was evaluated to examine whether observations from other lanes on the same link could support estimation for a masked target lane. The candidate pool consisted of 175 sensor-mapped lanes. Only links containing at least two observed lanes were eligible, ensuring that another observed lane remained available after masking the target lane. One target lane per selected link was randomly chosen using a fixed random seed. The reported analysis covered 34 road sections, comprising 13 two-lane, 3 three-lane, 14 four-lane, and 4 five-lane sections.

As shown in Table 6, the deep multimodal model achieved lower WMAPE than the average single-modal baseline across all lane-count groups. The largest gain occurred for four-lane sections, where WMAPE decreased from 9.41% to 8.05%, yielding a reduction of 1.36 percentage points (CI: [1.13, 1.61]). Gains for two-, three-, and five-lane sections were 0.29 (CI: [0.19, 0.40]), 0.60 (CI: [0.39, 0.81]), and 0.37 percentage points (CI: [0.29, 0.46]), respectively. These results highlight benefits within the evaluated sample, although the three- and five-lane comparisons remain exploratory because of their small sample sizes.

For four-lane links, additional analysis examined performance by lane position and movement type. The sample included four left-turn/inner, nine through, and one right-turn/outer lane sections. As shown in Table 7, the deep multimodal model reduced WMAPE from 8.98% to 7.01% for left-turn/inner lanes and from 10.23% to 8.99% for through lanes. The gain intervals were positive for both groups, ranging from 1.66 to 2.29 and from 0.98 to 1.53 percentage points, respectively. The right-turn/outer lane showed little difference, with WMAPE values of 3.81% and 3.78% for the deep multimodal and average single-modal models, respectively. These results indicate larger numerical benefits for the evaluated inner and through lanes, while the outer-lane result represents a single section. This variation suggests that lane position and movement type warrant consideration when assessing the benefits of multimodal integration.

**Figure 5:**
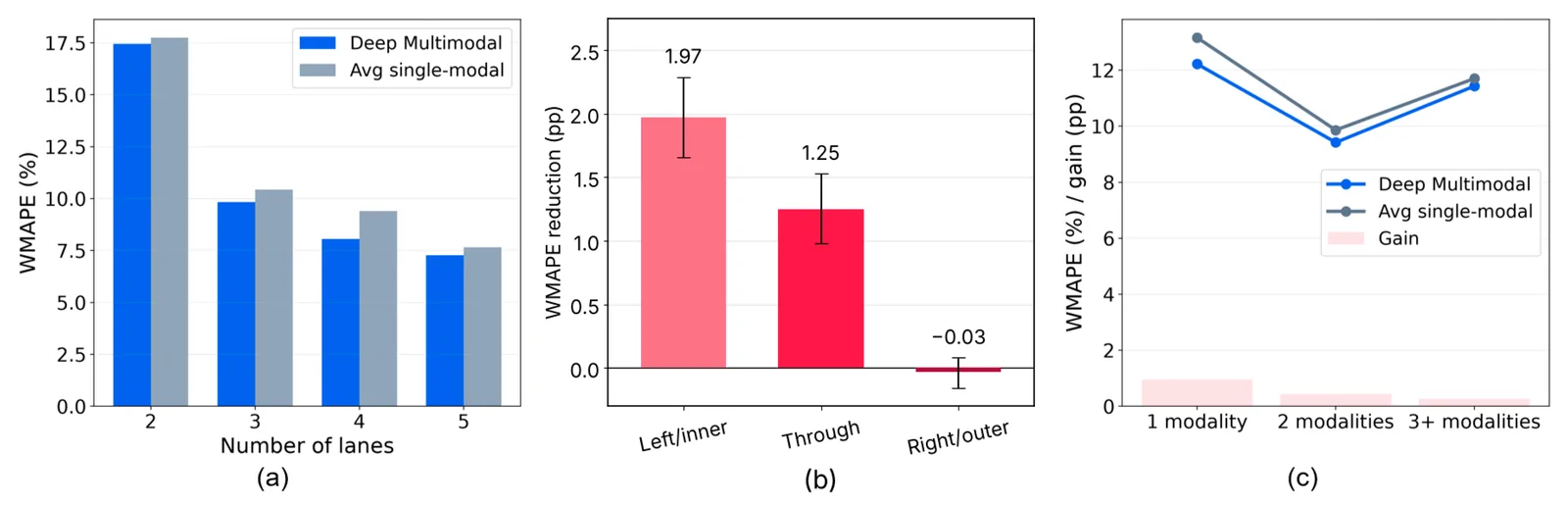
Lane-level sensitivity analysis: (**a**) WMAPE by number of lanes, (**b**) WMAPE reduction by lane position for 4-lane links, and (**c**) WMAPE by sensor availability.

The lane-level sensitivity results are further summarized in Figure 5. Figure 5a compares the deep multimodal model with the average single-modal baseline across different lane counts. Within the evaluated sample, the multimodal model shows lower WMAPE for all groups, with the largest reduction for 4-lane links. Because the number of sections in each group is small and lane count is not separated from other site characteristics, this pattern should be regarded as exploratory.

Figure 5b shows the WMAPE gain by lane position for 4-lane links, consistent with Table 7. The left-turn/inner and through lanes show gains of 1.97 and 1.25 percentage points, respectively, whereas the single right-turn/outer lane section shows little difference (−0.03 percentage points). These results suggest that multimodal information may be useful for lanes subject to turning and lane-changing interactions, although the evidence is limited to a small number of sections.

Figure 5c examines performance according to nearby sensor availability. Within the evaluated samples, the deep multimodal model shows lower WMAPE than the average single-modal baseline in all availability groups, with the largest gain when only one modality is directly available near the target link. Because each target sensor is also observed during training, this analysis does not represent estimation at uninstrumented locations; the result should be regarded as exploratory evidence that multimodal information may be most useful where local sensing coverage is limited.

Overall, the sensitivity analyses suggest that the benefit of multimodal sensing depends on both roadway configuration and sensor availability. The gains are larger in selected multi-lane settings and in locations with limited sensing coverage, highlighting the value of heterogeneous sensor integration for unevenly instrumented road networks.

### 4.5. Route-Cost Comparison

A route-cost comparison examined how the spatial resolution of estimated traffic speeds affects route selection and estimated travel costs. Link-level information assigns a representative speed to each road link, whereas lane-level information represents speed differences among individual lanes and movements. The comparison illustrates the influence of traffic-information resolution within the proposed multimodal framework.

Thirty-four OD pairs with network distances greater than 6 km were selected at the time step with the largest spatial speed variation. For each OD pair, minimum travel-time-based route costs were calculated using link-level and lane-level multimodal speed estimates. Both information levels used the same framework. The selected time step provides an illustrative comparison under spatially heterogeneous traffic conditions.

For each information level, the estimated speeds were converted into edge travel times by dividing the length of each link by the corresponding speed. In the lane-level formulation, each lane was represented as a directed edge with its own estimated speed and with connectivity defined by its lane-movement attribute (left-turn, through, or right-turn), whereas in the link-level formulation all lanes of a link were assigned the representative link speed. Because the network contains only three signalized intersections, signal delays and queueing effects were not modeled, and the minimum-cost route for each OD pair was obtained by a shortest-path search on the resulting network.

As summarized in Table 8, the mean minimum estimated route cost was 515.4 s (8.59 min) using link-level information and 477.7 s (7.96 min) using lane-level information. The difference was 37.7 s, corresponding to a 7.31% lower estimated cost under the lane-level formulation. Mean route distance increased from 7.333 km to 7.426 km, while the estimated route speed increased from 51.2 km/h to 56.0 km/h. These results show that incorporating lane-specific speeds can change selected routes and their estimated costs.

**Figure 6:**
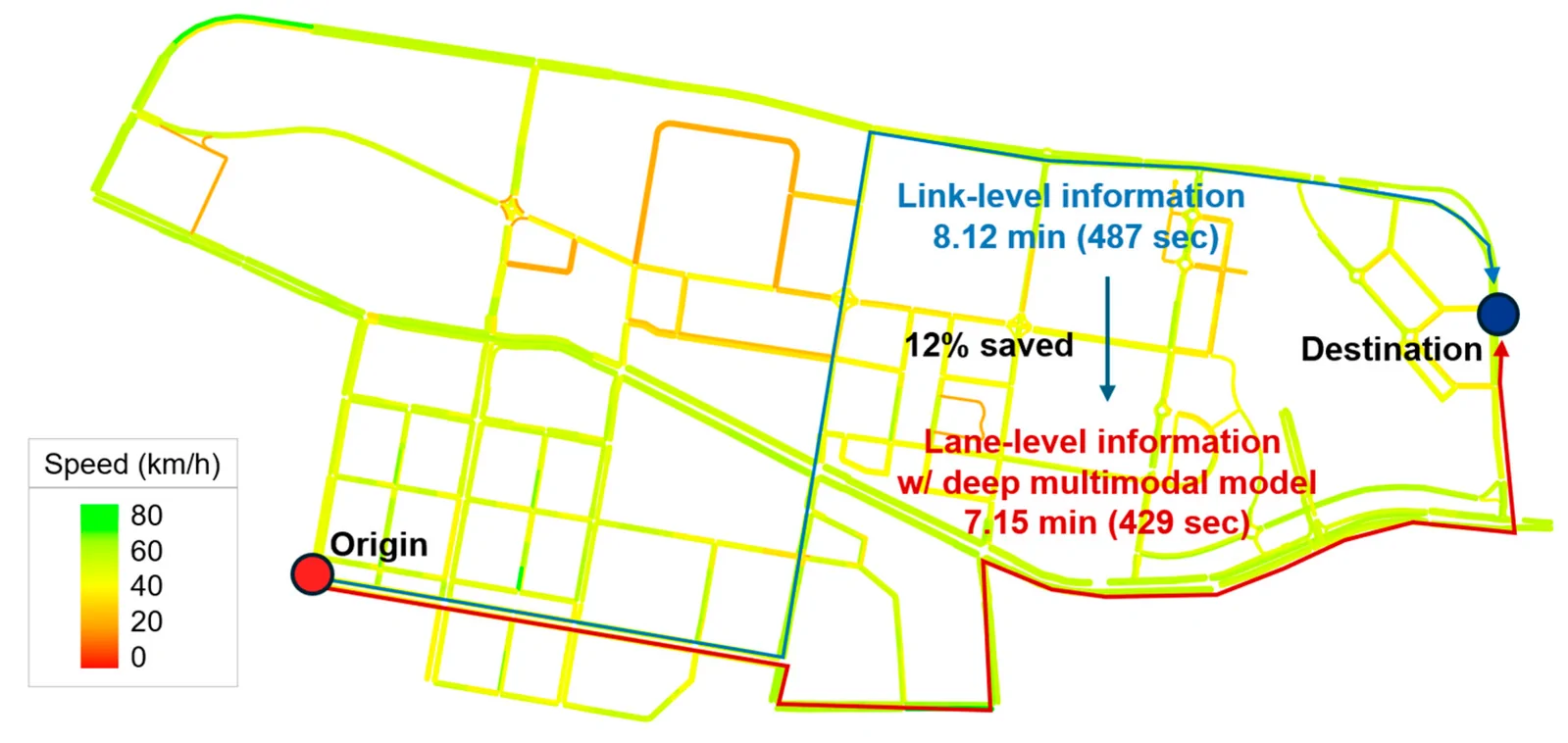
Example route comparison using link-level and lane-level traffic-speed information. The blue and red arrows trace the routes selected using link-level and lane-level estimates, respectively. The background road colors indicate speed (km/h).

Figure 6 illustrates one example, with estimated route costs of 8.12 min under the link-level formulation and 7.15 min under the lane-level formulation, corresponding to a difference of approximately 12%. This example demonstrates how lane-specific information can influence the representation of route costs. The results describe a model-based comparison, and operational travel-time benefits require independent validation.

## 5. Discussion

This section interprets the findings in relation to the three contributions of the study and to previous research rather than restating the numerical results. Overall, the results indicate that a single multimodal model can provide lane-level speed estimates comparable to or better than sensor-specific models, but the magnitude of the benefit varies across target sensor types, roadway conditions, and spatial contexts. These findings support the three main contributions of this study: heterogeneous sensor integration, lane-level traffic-state estimation, and missing observation estimation at existing sensor locations.

The first contribution is the integration of heterogeneous traffic sensing sources within a unified deep learning framework. Previous traffic forecasting and traffic-state estimation studies have demonstrated the importance of spatial relationships and multi-source information for improving network-level estimation [5,6,7,14,25,30,31,32,33,34,35]. The present results are consistent with this direction. The proposed framework extends previous approaches by jointly incorporating five sensing modalities with different measurement principles and spatial characteristics. The low correlations among VDS, CCTV, LiDAR, RTMS, and AVC indicate that the observed speeds differ substantially across sensing sources, although these differences may also reflect deployment locations and measurement characteristics. The cross-modal results further support this interpretation. Each single-modal model generally performed best for its corresponding sensor type, but its performance deteriorated for other target modalities. This pattern is consistent with previous studies showing that sensing technologies differ in spatial resolution, observable variables, and environmental sensitivity [22,23,24,25,26,27]. Multimodal integration therefore provides a mechanism to combine complementary information and reduce dependence on the limitations of any single sensing source [25,31,32,33,34,35].

The second contribution is lane-level traffic-speed estimation. Previous traffic-state studies have often focused on link-level or aggregated traffic conditions [8,9,10,28,29,30], but recent research has increasingly emphasized lane-level information for capturing detailed traffic dynamics in multi-lane and complex roadway environments [11,12,13,14,15,16,17,18,19,20,21]. The results of this study are consistent with these studies. The multimodal gain differed according to lane configuration and lane position, and the largest improvement was observed for 4-lane links. For 4-lane links, the left-turn/inner and through lanes showed larger improvements than the single right-turn/outer lane section, although these lane-position results are exploratory. These results suggest that lane-level estimation becomes more important where localized traffic interactions cannot be adequately represented by an average link speed. Previous lane-level studies have similarly shown that traffic states can vary substantially across adjacent lanes because of lane-specific flow patterns, vehicle interactions, and spatial dependencies [11,12,13,15,16,17,18,19,20,21]. The proposed framework extends this direction by estimating lane-level conditions using heterogeneous infrastructure sensing rather than relying on a single observation technology.

The third contribution is missing observation estimation at existing sensor locations. Traffic observations can be unavailable because a sensor is not installed, temporarily fails, or has limited spatial coverage. Previous studies have addressed incomplete traffic information using probe vehicles, fixed-mobile sensor fusion, interpolation, and heterogeneous data fusion [28,29,30,31,32,33,34,35]. Multimodal learning has also been applied to estimate traffic speed using complementary sensing sources [35]. The proposed framework extends these approaches by using information from surrounding sensor-equipped links and multiple sensing modalities to estimate traffic conditions at target locations with limited or unavailable direct observations. The model considers graph proximity, Euclidean proximity, bearing similarity, and intersection similarity rather than relying only on immediately adjacent links. This is consistent with previous studies showing that traffic conditions can be influenced by broader network relationships [5,6,7,14,19,20,21]. The sensor-availability analysis also showed that the multimodal gain was largest when only one sensing modality was directly available near the target location. This exploratory finding suggests that multimodal learning may be particularly valuable in sparsely instrumented sections of the road network, whereas estimation at locations where no sensor has been installed should be examined through sensor-, link-, or region-disjoint experiments.

From an operational perspective, the proposed framework supports continuous lane-level traffic monitoring across networks with uneven sensor coverage. Detailed traffic-state information is relevant to traffic management, route guidance, and broader intelligent transportation applications [39,40,41,42,43,44,45,46,47,48,49]. Its value is particularly high near intersections and route-choice points, where localized congestion influences diversion decisions. Estimating traffic states at sensor locations with missing observations can provide information before vehicles enter congested sections. The practical benefit of multimodal sensing therefore extends beyond estimation accuracy and contributes to more detailed and spatially continuous traffic information across heterogeneous urban road networks.

## 6. Conclusions

This study developed a deep multimodal learning framework for lane-level traffic-speed estimation using five heterogeneous sensing sources: VDS, CCTV, LiDAR, RTMS, and AVC. The proposed model integrates sensor-specific dynamic features, static roadway characteristics, spatial relationships, and modality availability within a unified architecture. The model was evaluated against five single-modal baselines across different time steps, target sensor types, and lane-level roadway conditions. The results show that the deep multimodal model provided accuracy comparable to or better than the matched single-modal models within a single unified model. Its pooled WMAPE remained below 10% across the evaluated temporal aggregation intervals, while target-specific WMAPE ranged from 2.76% to 20.76%. Compared with the matched single-modal models, the multimodal model reduced WMAPE for CCTV and AVC targets and performed comparably for VDS, LiDAR, and RTMS targets. The lane-level analysis further suggested that multimodal gains may vary by roadway configuration and sensor availability, although these results are exploratory because of the limited number of evaluated lane sections.

The main contribution of this study is threefold. First, the framework jointly integrates five traffic sensing modalities and learns complementary information across heterogeneous sensing technologies. Second, it estimates traffic conditions at the lane level, providing more detailed information than conventional link-level approaches. Third, it enables lane-level speed estimation at existing sensor locations when their observations are unavailable, using information from surrounding links and other available sensing modalities. This capability can reduce dependence on dense deployment of a single sensing technology and support more continuous traffic monitoring across unevenly instrumented road networks. The proposed framework supports applications requiring detailed and spatially continuous traffic information, including traffic management, route guidance, and automated vehicle systems. Lane-level traffic-state estimation is useful near intersections, route-choice points, and sections with limited sensing coverage.

Future research should extend the framework to larger and more diverse road networks and evaluate its transferability under different traffic and sensor deployment conditions. Controlled sensor-removal experiments and sensor-, link-, or region-disjoint evaluations could be used to quantify missing observation estimation performance under progressively reduced sensing coverage. Future work should also examine dynamic sensor failures and identify the minimum sensing configuration required for reliable network-wide estimation. In addition, the estimated lane-level traffic states can be integrated with route-choice models to evaluate whether improved multimodal traffic information leads to better diversion decisions and measurable travel-time savings.

## Figures and Tables

**Table 1:** The descriptive statistics of the processed data.

Sensor	Sites	Speed Mean (km/h)	Speed Std. (km/h)
VDS	119	38.41	20.76
CCTV	55	12.11	9.89
LiDAR	67	24.53	12.09
RTMS	33	8.08	3.28
AVC	150	60.51	35.86

**Table 2:** Overall performance of the deep multimodal and single-modal models.

Model	Time Step	WMAPE (%)	MAE	RMSE
Deep Multimodal	5 min	8.22	5.86	7.70
	15 min	8.89	6.16	7.85
	30 min	9.13	6.52	7.70
	1 h	9.66	6.65	9.56
	2 h	9.71	6.70	9.82
single-VDS	5 min	9.27	6.61	7.32
	15 min	9.96	7.10	9.06
	30 min	11.12	7.54	11.32
	1 h	11.40	7.90	10.21
	2 h	11.43	7.88	11.77
single-CCTV	5 min	20.04	14.30	16.43
	15 min	22.18	15.82	18.13
	30 min	22.41	15.52	17.49
	1 h	22.77	15.45	17.49
	2 h	24.07	16.60	18.74
single-LiDAR	5 min	13.84	9.58	11.27
	15 min	14.85	10.07	12.34
	30 min	15.03	10.71	12.17
	1 h	15.14	10.44	12.74
	2 h	17.63	12.58	13.58
single-RTMS	5 min	11.05	7.89	10.01
	15 min	11.14	7.95	9.69
	30 min	11.31	7.83	9.64
	1 h	11.96	8.11	11.18
	2 h	12.59	8.68	12.08
single-AVC	5 min	10.29	7.34	9.13
	15 min	10.89	7.77	10.35
	30 min	10.96	7.79	10.48
	1 h	11.44	7.89	10.74
	2 h	11.26	7.64	10.42

**Table 3:** Ablation results of the deep multimodal model.

Model	WMAPE (%)
Full model	8.92 ± 1.01
Without Transformer	9.49 ± 1.11
Without spatial bias	9.07 ± 0.70
Simple fusion	11.44 ± 1.05

**Table 4:** Computational settings and inference latency of the full model.

Item	Setting/Result
CPU	AMD Ryzen 7 9800X3D
GPU	NVIDIA RTX 5070 Ti, 16 GB
Framework/Precision	PyTorch 2.7.1/FP16
Network size	821 links
Estimation workload	4105 queries: 821 links × 5 modalities
Batch size	256
GPU inference, mean/P95	35.68/37.77 ms
Processing latency, mean/P95	47.98/51.08 ms

**Table 5:** Sensor-specific WMAPE performance of deep multimodal and baseline models.

Target Sensor	Deep WMAPE (%)	Matched Single WMAPE (%)	Gain, pp (95% CI)
VDS	2.76	2.64	−0.12 [−0.43, 0.22]
CCTV	20.76	21.67	+0.91 [0.12, 1.89]
LiDAR	9.60	9.40	−0.20 [−0.73, 0.27]
RTMS	8.21	8.07	−0.13 [−1.41, 1.10]
AVC	10.20	11.38	+1.18 [−0.08, 2.13]

**Table 6:** Lane-level performance by number of lanes.

Number of Lanes	Lane Sections	WMAPE (%)		Gain (pp)
		Deep	Single	
2	13	17.46 [16.56, 18.53]	17.75 [16.82, 18.89]	0.29 [0.19, 0.40]
3	3	9.84 [9.00, 10.73]	10.44 [9.60, 11.35]	0.60 [0.39, 0.81]
4	14	8.05 [7.37, 8.76]	9.41 [8.53, 10.34]	1.36 [1.13, 1.61]
5	4	7.28 [6.96, 7.61]	7.65 [7.31, 8.01]	0.37 [0.29, 0.46]

**Table 7:** Performance by lane position for 4-lane links.

Lane Position	Lane Sections	WMAPE (%)		Gain (pp)
		Deep	Single	
Left-turn/inner lane	4	7.01 [6.47, 7.58]	8.98 [8.21, 9.78]	1.97 [1.66, 2.29]
Through lanes	9	8.99 [8.17, 9.86]	10.23 [9.19, 11.34]	1.25 [0.98, 1.53]
Right-turn/outer lane	1	3.81 [3.46, 4.17]	3.78 [3.46, 4.09]	−0.03 [−0.16, 0.08]

**Table 8:** Route-cost comparison using link-level and lane-level speed estimates.

Metric	Link-Level Speed	Lane-Level Speed
Distance (km)	7.333	7.426
Minimum estimated route cost (s)	515.4	477.7
Minimum estimated route cost (min)	8.59	7.96
Route speed (km/h)	51.2	56.0
Reduction	-	7.31%

## Data Availability

The data used in this study were provided by a third party and are not publicly available due to data-sharing restrictions.

## Abbreviations

The following abbreviations are used in this manuscript:

ARIMA	Autoregressive Integrated Moving Average
AVC	Automatic Vehicle Classification
CCTV	Closed-Circuit Television
DSRC	Dedicated Short-Range Communication
GELU	Gaussian Error Linear Unit
LiDAR	Light Detection and Ranging
MAE	Mean Absolute Error
MLP	Multilayer Perceptron
OD	Origin–Destination
RMSE	Root Mean Squared Error
RTMS	Remote Traffic Microwave Sensor
VDS	Vehicle Detection System
WMAPE	Weighted Mean Absolute Percentage Error
